# Hernie de Littré ombilicale étranglée chez l’enfant: complication rare d’une malformation fréquente de l’intestin grêle

**DOI:** 10.11604/pamj.2018.30.214.14486

**Published:** 2018-07-17

**Authors:** Sourou Bruno Noukpozounkou, Ismaïl Lawani, Ogounrila Thomas Armel Elegbede, Djifid Morel Seto, Beaudelaire Romulus Assan, Amoussou Sèdjro Clotaire Roméo Houegban, Houenoukpo Koco, Michel Armand Fiogbe

**Affiliations:** 1 Clinique Universitaire de Chirurgie Pédiatrique CNHU-HKM, Cotonou, Bénin; 2 Département de Chirurgie, Faculté des Sciences de la Santé, Université d’Abomey-Calavi, Cotonou, Bénin

**Keywords:** Hernie ombilicale, hernie de Littré, diverticule de Meckel, Umbilical hernia, Littré's hernia, Meckel's diverticulum

## Abstract

Le diverticule de Meckel est l'anomalie congénitale la plus fréquente de l'intestin grêle. Bien qu'il s'agisse d'une anomalie courante dans la population générale, sa présence dans un sac herniaire, en particulier au niveau ombilical est une situation peu fréquente et constitue la hernie de Littré. Nous rapportons le cas d'un nourrisson de 6 mois de sexe féminin, admis pour une tuméfaction ombilicale douloureuse et irréductible. Le diagnostic de hernie ombilicale étranglée a été posé. En per opératoire, il était découvert dans le sac herniaire un diverticule de Meckel inflammatoire. On procéda à une résection cunéiforme du diverticule suivie de la fermeture de la brèche intestinale par une suture en surjet et de la réfection pariétale. Le diagnostic clinique de la hernie de Littré est difficile et l'attitude thérapeutique varie selon les équipes.

## Abstract (en)

Meckel's diverticulum is the most common congenital anomaly of the small bowel. Although it is a common anomaly in the general population, its occurrence in a hernial sac, in particular at the umbilicus, is uncommon and it is known as Littré's hernia. We here report the case of a 6-month female infant, admitted with painful irreducible umbilical swelling. The diagnosis of strangled umbilical hernia was made. Meckel inflammatory diverticulum in the hernial sac was detected intraoperatively. The patient underwent wedge resection of the diverticulum followed by the closure of the intestinal gap using continuous suture and parietal repair. Clinical diagnosis of Littré's hernia is difficult and therapeutic approach varies depending on the medical team.

## Introduction

La hernie de Littré est définie comme la présence du diverticule de Meckel dans un sac herniaire [1]. Il a été décrit pour la première fois par Alexis Littré en 1700; cependant le terme de hernie de Littré a été utilisé pour la première fois par Reinke en 1841 [2]. Chez l'enfant, la hernie de Littré siège le plus souvent dans l'ombilic, et peut être le siège d'une hétérotopie gastrique ou pancréatique [3]. Nous rapportons ici un cas de hernie de Littré ombilicale étranglée associée à une malformation ano-rectale, qui nous permet de faire une revue de littérature sur la question.

## Patient et observation

**Figure 1:**
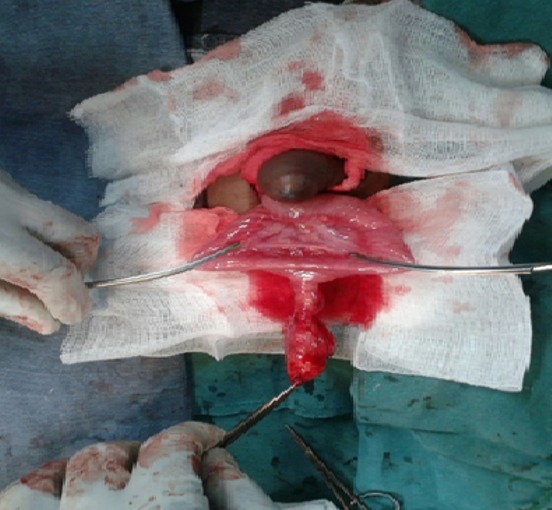
Vue per opératoire du diverticule de Meckel

Il s'agissait d'un nourrisson de 24 semaines, de sexe féminin, amené par les parents en consultation pour une tuméfaction douloureuse de l'ombilic. L'interrogatoire a retrouvé un début 24 heures environ avant admission par des pleurs incessantes exacerbés au toucher de l'ombilic, associés secondairement à une fièvre non chiffrée. Il n'y avait pas de signes d'occlusion, ni d'hémorragie digestive, mais une malformation ano-rectale avec fistule recto-vestibulaire était associée. L'examen physique initial avait retrouvé: un bon état général, un syndrome infectieux (hyperthermie à 39,1 degrés, tachycardie à 102 pulsations par minutes), une tuméfaction ombilicale non expansive à la toux, irréductible et douloureuse avec un maximum de douleur au collet. Les autres orifices herniaires étaient libres. Le diagnostic de hernie ombilicale étranglée a été retenu. Les tentatives de réduction par taxis furent vaines, indiquant l'intervention chirurgicale. En per opératoire, à l'ouverture du sac herniaire, on y avait découvert un diverticule de Meckel inflammatoire (Figure 1). On a procédé à une résection cunéiforme du diverticule, une suture en surjet de la brèche intestinale et une réfection pariétale par des points de fil résorbable. Les suites opératoires ont été simples, l'examen histologique n'a pas montré de tissus hétérotopique dans le diverticule.

## Discussion

Le diverticule de Meckel est l'anomalie congénitale la plus fréquente de l'intestin grêle. Sa fréquence dans la population globale est estimée à 2% [3]. Il est lié à une anomalie de fermeture du canal omphalo-mésentérique [4]. Il contient toutes les couches de l'intestin normal [5]. Il contient quelques fois de la muqueuse ectopique, gastrique ou pancréatique [3]. Il est localisé sur le bord anti-mésentérique de l'iléon à 30 à 90 cm environ de la valvule iléo-caecale, mesurant 3-6 cm de long et 2 cm de diamètre [6]. Dans notre cas le diverticule de Meckel mesurait 6 cm de long et était situé à 40 cm environ du carrefour iléo-caecal. Au cours de son développement dans la cavité péritonéale, le diverticule a tendance de se déplacer vers les zones de faiblesse de la paroi abdominale et à adhérer au fond d'un sac herniaire constituant ainsi la hernie de Littré. Ce type de hernie a été décrit pour la première fois suite à des constatations d'autopsie de deux malades par Alexis Littré ayant objectivé un diverticule iléal dans le sac d'une hernie inguinale. L'ombilic est le siège le plus fréquent de la hernie de Littré chez l'enfant dans 85% des cas [7]. Bien que le diverticule de Meckel soit une anomalie courante dans la population générale, sa présence dans un sac herniaire est une situation peu fréquente [6]. Et sa traduction clinique est exceptionnelle. Le diagnostic clinique d'une hernie de Littré est difficile. Seulement 4 à 6% des cas de diverticule de Meckel sont cliniquement symptomatiques. Chez l'enfant, la symptomatologie est habituellement faite d'un saignement gastro intestinal indolore à partir de 2 ans de vie [8]. La réduction incomplète d'une hernie étranglée, la présence d'une fistule entéro cutanée à travers le sac de la hernie, l'existence d'un saignement rectal sont les seuls éléments cliniques pouvant orienter vers une hernie de Littré [9]. L'étranglement, la nécrose et la perforation sont des complications rares chez l'enfant. Le diagnostic de hernie de Littré est le plus souvent fait en per opératoire. Dans notre cas aucun élément clinique n'avait permis d'évoquer ce diagnostic. Le tissu ectopique est présent dans la muqueuse du diverticule jusqu'à hauteur de 80% lorsqu'il est symptomatique [3]. Dans notre cas, l'examen anatomo-pathologique n'avait pas retrouvé de tissu ectopique.

Sur le plan thérapeutique, il y a deux situations: la situation du diverticule de Meckel symptomatique et celle du diverticule asymptomatique découverte de façon fortuite. Dans la situation du diverticule de Meckel symptomatique, la résection chirurgicale est la règle [3]. Elle peut consister soit en une excision simple du diverticule ou en une résection iléale emportant le diverticule, suivie d'une anastomose iléo-iléale termino-terminale [5]. Le choix de cette seconde technique est soustendu par la possibilité de tissu ectopique qui s'étend au-delà du diverticule. Notre patiente avait bénéficié d'une résection du diverticule suivie d'une suture. De nos jours la mortalité liée à cette intervention est très basse [3]. Dans la situation du diverticule de Meckel asymptomatique, l'attitude thérapeutique est controversée du fait que les complications futures d'un diverticule de Meckel non réséqué doivent contre peser les complications post opératoires d'une résection qui sont de l'ordre de 9% dans la série de Soltero et Bill et de 6% dans la série de Morover [10]. Les attitudes sont variables selon les équipes chirurgicales allant d'une abstention à une résection. La grande taille (6cm) du diverticule de notre patient était un critère supplémentaire justifiant sa résection. En effet, les difficultés thérapeutiques ont permis d'identifier les critères prédictifs pour qu'un diverticule de Meckel asymptomatique devienne symptomatique, et qui justifient une résection systématique. Il s'agit de sa longueur supérieure à 2 cm, du sexe du patient (surtout masculin), du jeune âge du patient et de la présence d'une bande fibreuse de tissu ectopique [11]. L'association d'un diverticule de Meckel symptomatique et d'une malformation ano-rectale n'a pas été retrouvée dans la littérature.

## Conclusion

La hernie de Littré étranglée est une complication rare du diverticule de Meckel. Son diagnostic se fait habituellement en per opératoire. La résection chirurgicale du diverticule est systématique en présence de signes cliniques. En cas de découverte fortuite, l'attitude thérapeutique est variable en fonction des équipes.

## Conflits d’intérêts

Les auteurs ne déclarent aucun conflit d'intérêts.
